# Megacystis Microcolon Intestinal Hypoperistalsis Syndrome (MMIHS): A Rarity

**Published:** 2015-01-10

**Authors:** Naeem Liaqat, Sajid Nayyar, Asif Iqbal, Sajid Hameed Dar

**Affiliations:** Department of Paediatric Surgery, SIMS/ Services Hospital, Lahore, Pakistan

**Dear Sir**

A 2-day-old male neonate, born full term through Caesarian section, presented to our department with bilious vomiting since birth and unable to pass meconeum. On examination, the patient had distended abdomen. A mass was palpable in the hypogastrium and left iliac region. His bowel sounds were not audible; rectal examination showed no meconium. His baseline investigations were within normal ranges. Abdominal radiograph showed few gas shadows. Ultrasound of the abdomen was unremarkable except for a large cystic mass in pelvis which was ‘megacystis’ according to radiologist. Upon exploration, a large fluid containing mass was encountered occupying whole left side of the abdomen and was pushing intestine towards right (Fig.1). The mass was the distended urinary bladder with dilated and tortuous both ureters. Intestine of patient was malrotated with multiple adhesion bands. Caliber of distal small intestine, caecum and large intestine were micro sized. However whole length of intestine was patent and no atresia was identified. On the basis of these findings the diagnosis of Megacystis Microcolon Intestinal Hypoperistalsis (MMIHS) was made and an ileostomy was made. Postoperatively total parenteral nutrition was provided to the patient. The biopsies taken from ileostomy site, ascending colon and sigmoid colon, all showed normal ganglion cells. The patient is now 1 month old and is still on total parenteral nutrition and prokinetics.

**Figure:**
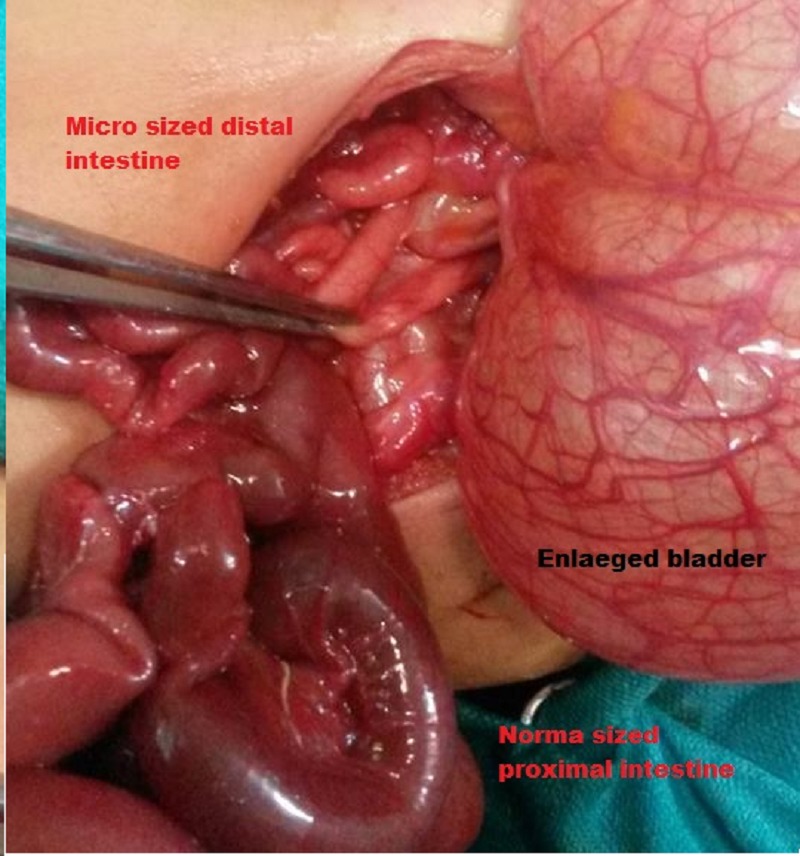
Figure 1: Distended urinary bladder and disparity in size between proximal and distal intestines.

MMIHS is a rare cause of neonatal intestinal obstruction with around 230 cases reported in the literature. [1-3] It was first described by Berdon and colleagues in 1976. [2] Females are 3 - 4 times more commonly affected than males. Its association with consanguinity has been found and it is thought to have an autosomal recessive inheritance. [1] Etiology of MMIHS is unclear and different hypothesis have been proposed including neurogenic, myogenic, genetic and hormonal origin. In most of the patients histologically normal ganglion cells are found; however, in some deficient ganglion cells have also been reported. Puri and colleagues had proposed myogenic origin of this disease and found decreased smooth muscle in wall of intestine. [1] In another study deficient interstitial cells of Cajal have also been reported in intestine and urinary bladder. [4] Prenatal diagnosis had been done in few reported cases in literature on account of distended urinary bladder, hydronephrosis and variation of amniotic fluid level. [5] Generally patients with MMIHS have poor outcome and need long term parenteral nutrition and prokinetics. Some of patients need vesicostomy; however, there is no consensus of doing vesicostomy at initial presentation.

## Footnotes

**Source of Support:** Nil

**Conflict of Interest:** None

